# Genistein and Aphid Probing Behavior: Case Studies on Polyphagous Aphid Species

**DOI:** 10.3390/molecules29235715

**Published:** 2024-12-03

**Authors:** Anna Wróblewska-Kurdyk, Bożena Kordan, Katarzyna Stec, Jan Bocianowski, Beata Gabryś

**Affiliations:** 1 Department of Botany and Ecology, University of Zielona Góra, Szafrana 1, 65-516 Zielona Góra, Poland; a.wroblewska@wnb.uz.zgora.pl (A.W.-K.);; 2 Department of Entomology, Phytopathology and Molecular Diagnostics, University of Warmia and Mazury in Olsztyn, Prawocheńskiego 17, 10-720 Olsztyn, Poland; bozena.kordan@uwm.edu.pl; 3 Department of Mathematical and Statistical Methods, Poznań University of Life Sciences, Wojska Polskiego 28, 60-637 Poznań, Poland; jan.bocianowski@up.poznan.pl

**Keywords:** black bean aphid, peach-potato aphid, bird cherry-oat aphid, plant allelochemicals, flavonoids, deterrents

## Abstract

(1) Background: Genistein is a naturally occurring flavonoid with a rich spectrum of biological activities, including plant-herbivore interactions. The aim of the study was to evaluate the effect of exogenous application of genistein on aphid behavior during probing in plant tissues. (2) Methods: *Vicia faba*, *Brassica rapa* ssp. *pekinensis*, and *Avena sativa* were treated transepidermally with a 0.1% ethanolic solution of genistein, and the probing behavior of generalist aphid species *Aphis fabae*, *Myzus persicae*, and *Rhopalosiphum padi* was monitored on their respective treated and untreated host plants using electropenetrography (=electrical penetration graph technique, EPG); (3) Results: Genistein did not deter aphid probing activities in non-phloem tissues. In *A. fabae* and *R. padi*, a trend towards reduction and in *M. persicae* a trend towards increase in phloem sap ingestion occurred on genistein-treated plants, but these trends were not statistically significant. (4) Conclusions: Genistein is not a deterrent chemical against generalist aphid species studied; therefore, it is not recommended for practical application.

## 1. Introduction

**Figure 1:**
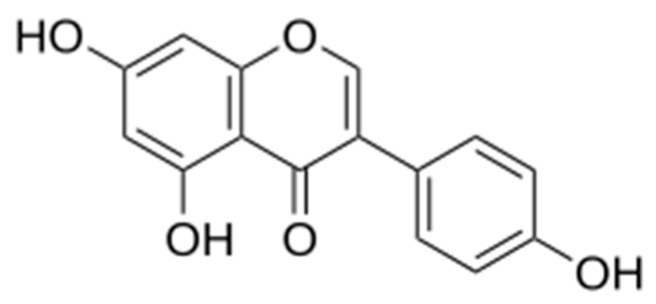
Chemical structure of genistein [2].

Genistein, the 5,7-dihydroxy-3-(4-hydroxyphenyl)chromen-4-one (Figure 1), is a naturally occurring flavonoid constituent mainly known from the legume plants (Fabaceae), isolated for the first time in 1899 from the dyer’s broom, *Genista tinctoria* L. (Fabaceae) [1,2].

Since the discovery, a great deal of data have been accumulated that demonstrate the extraordinary biological activity of this compound. The best-known activities are the therapeutic effects of genistein, which include its antioxidant activity, inhibition of inflammation, estrogenic properties, prophylaxis and treatment of cancer, and various chronic diseases such as atherogenic condition, hypercholesterolemia, and diabetes [3,4,5]. Genistein is also toxic to mosquito *Aedes aegypti* (L.) (Diptera: Culicidae) larvae [6] and has anthelmintic properties [7]. However, the fundamental function of genistein, as a plant secondary compound, is the participation in specialized plant physiological and metabolic functions and plant responses to stresses, such as changes in light or temperature, competition, herbivore pressure, and pathogenic attack [8,9]. Genistein also participates in the initiation of the symbiosis of legumes with mycorrhizal fungi and nitrogen-fixing bacteria as a signaling molecule [9].

In view of its broad and potent biological activity, the application of genistein as a prospective natural compound in plant pest control has also been considered, including the engineering of plants for pest resistance [10]. This idea is supported by the evidence that herbivores and plant stress-related elicitor cis-jasmone induce an increase in genistein content in the plants, which, in turn, may reduce the performance of herbivores [11,12].

The research on genistein-insect-herbivore interactions carried out hitherto involved species of different systematic groups representing different life histories and modes of feeding. These studies demonstrated a high species specificity of effects on various aspects of insects’ lives. Genistein induced an increase in hemocyte concentration in *Periplaneta americana* (L.) (Blattodea: Blattidae), while the total hemocyte count in *Schistocerca gregaria* Forsskål (Orthoptera: Acrididae) was significantly suppressed and only moderately altered in *Dysdercus cingulatus* (Fabricius) (Hemiptera: Pyrrhocoridae) [13]. In *Tribolium castaneum* (Herbst) (Coleoptera: Tenebrionidae), genistein appeared cytotoxic, which caused a decrease in the survival of adults [14]. When added to the diet, genistein significantly decreased the number of progeny of the Formosan subterranean termite, *Coptotermes formosanus* Shiraki (Isoptera: Rhinotermitidae) [15]. The growth rate, body mass, and overall biological performance of *Oedaleus asiaticus* Bey-Bienko (Orthoptera: Acrididae) were reduced significantly following the treatment with genistein; therefore, this compound was recommended for use in a biopesticide formulation for insect pest control in grasslands and other crop plants as a potential application to grasshopper biological control [16]. In contrast, genistein did not affect the survivorship, growth, or fecundity of *Lymantria dispar* (L.) (Lepidoptera: Erebidae); therefore, it was not recommended as a deterrent against generalist tree foliar herbivores [17]. Plants, on the other hand, respond to herbivore infestation also in an herbivore- and plant-species-specific manner. This was shown in soybean, *Glycine max* (L.) Merr. and *Caliothrips phaseoli* (Hood) (Thysanoptera: Thripidae) and *Spodoptera frugiperda* (J. E. Smith) larvae (Lepidoptera: Noctuidae) that differ in the way of feeding. The feeding of *C. phaseoli*, which possesses sucking-piercing mouthparts, caused an increase in malonyl genistein and genistein content in infested plants, while the feeding of *S. frugiperda*, which uses the chewing mouthparts, did not [18]. Interestingly, the content of genistein in infested plants may turn out highly variable: another soybean herbivore, the silverleaf whitefly, *Bemisia tabaci* (Gennadius) (Hemiptera: Aleyrodidae), causes an increase or decrease in the content of genistein, depending on the soybean cultivar and phenological stage [19].

The role of genistein in aphid (Hemiptera: Aphididae)-plant relationships has also been investigated, considering both the plant response to aphid infestation and aphid reaction to plants with various contents of this flavonoid as well as to the pure compound. As in other insect groups, the examples of which were provided earlier, the results of these studies are highly case-specific. Genistein in the leaves of soybeans infested by cowpea aphids, *Aphis craccivora* Koch (Hemiptera: Aphididae), was induced to higher contents post-infestation, and the activity of enzymes associated with the biosynthesis of flavonoids in the aphid-infested leaves strongly increased, which was proportional with the induced increase in genistein [20]. However, when soybean was infested by the soybean aphid *Aphis glycines* Matsumura, genistein levels increased in the resistant but not the susceptible genotypes [21]. Another study showed that only the long-term colonization of soybean by the soybean aphid caused an increase in genistein also in susceptible cultivars, but when applied to the cut plant leaves in dimethyl sulfoxide (DMSO) solution at a concentration of 5 µg/mL, genistein did not affect the behavior of *A. glycines* in the choice test [22]. Yet other studies on *A. glycines* and soybean cultivars demonstrated that there was a low correlation between the number of aphids per leaf, feeding damage, and leaf genistein levels [23]. In contrast, genistein was significantly upregulated in both resistant and susceptible cultivars of sorghum, *Sorghum bicolor* (L.) Moench (Poaceae), infested by the sorghum aphid *Melanaphis sacchari* (Zehntner) (Hemiptera: Aphididae), and the spraying of plants with 0.1% genistein improved the sorghum’s resistance to *M. sacchari* feeding [24]. The deterrent activity of genistein against the pea aphid *Acyrthosiphon pisum* (Harris) (Hemiptera: Aphididae) was demonstrated when it was included in the artificial diet at a relatively high concentration of ≥1000 µg/cm^3^ [25]. In another study, it was hypothesized that the resistance of soybean to the pea aphid was associated with high contents of genistein in soybeans’ vegetative parts [26]. This view was verified when the pea aphid’s preferred host plants, the pea *Pisum sativum* L. (Fabaceae), were treated transepidermally with 0.1% genistein solution: the probing behavior of *A. pisum* was not affected [27]. However, in alfalfa, *Medicago sativa* L. (Fabaceae), the increase in genistein confers resistance against the *Pisum* host race of *A. pisum* [11].

The high specificity in reciprocal responses of plants and phytophagous insects involved in direct trophic relationships should certainly be taken into account when considering the use of plant allelochemicals in crop pest control management practices. The studies on genistein-aphid relationships carried out hitherto have concentrated mainly on plants with high constitutive levels of genistein, the legumes, and aphid species that specialize to feed on these plants: the soybean aphid, the cowpea aphid, and the pea aphid, with the sorghum aphid being an exception [11,20,21,22,23,24,25,26,27]. The diverse spectrum of genistein effects on herbivorous insects [11,12,13,14,15,16,17,18,19,20,21,22,23,24,25,26,27] motivated us to carry out studies on polyphagous aphid species that infest a broad range of host plants: the black bean aphid *Aphis fabae* Scopoli, the peach–potato aphid *Myzus persicae* (Sulz.), and the bird cherry-oat aphid *Rhopalosiphum padi* (L.). The rationale of our research was to explore the prospects of using genistein as an anti-aphid agent in plant protection. Aphids reduce crop yield directly by removing nutrients from plant transporting vessels and indirectly as very efficient vectors of plant virus diseases [28,29,30,31]. In general, the direct damage due to aphid feeding is estimated at approximately 2% of all damage due to insect herbivores [32]. At the same time, the losses due to virus diseases transmitted by aphids may exceed the level of direct damage many times [33,34]. Aphids are vectors of nearly 50% of insect-borne viruses [31]. We concentrated our study on *A. fabae*, *M. persicae*, and *R. padi* that are listed among the 15 aphid species of most agricultural importance worldwide: the black bean aphid is the major pest of leguminous plants but also infests other important crops, such as sugar beet; the bird cherry-oat aphid attacks all major cereals and grasses; and the peach–potato aphid is extremely polyphagous and an exceptionally highly efficient virus vector whose mechanisms of insecticide resistance are the most diverse [28,29,35]. At present, direct control of aphids depends mainly on the use of neurotoxic insecticides. Considering the trend of eliminating such pesticides due to their broad-spectrum toxicity towards non-target organisms and the potential threat to humans and the environment, we investigated the possibility of using genistein as a species-specific aphid behavior-modifying chemical of negligible environmental impact. The influence of genistein on the instinctive behavioral traits in host plant selection by *A. fabae*, *M. persicae*, and *R. padi* has never been studied.

The aim of the present study was to determine the effect of exogenous application of genistein to the plant surface on aphid probing behavior. The probing behavior embraces all activities of aphid piercing–sucking mouthparts within plant tissues. These activities include the progressive movements of the piercing stylets within plant tissues to the basic food source of aphids, the plant phloem, and the actual feeding, i.e., the ingestion of phloem sap from sieve elements [30,36]. As aphids are able to respond to plant chemistry while penetrating both the non-phloem and phloem tissues, the monitoring of their probing behavior is crucial for the understanding of mechanisms of plant resistance against aphids and the interpretation of the allelochemicals’ bioactivity [37,38,39,40]. The aphid stylets’ movements inside the plant are unavailable for direct observation; therefore, we applied the electropenetrography (=electrical penetration graph, EPG, technique) in our study, which visualizes aphid activities in plant tissues, thus making them open for interpretation [30,36]. The effect of any unusual situation at the aphid–plant interface is manifested as a deviation from the typical behavioral model and can be evaluated based on parameters derived from the EPG recordings [37,38,39,40,41].

## 2. Results

**Figure 2:**
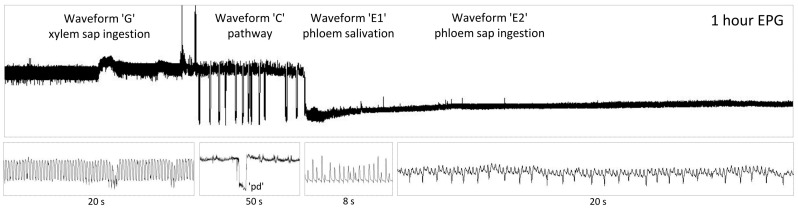
Visualization of aphid stylet activities in plant tissues recorded using electropenetrography (a sample derived from EPG recording of *Myzus persicae* on *Brassica rapa* ssp. *pekinensis* treated with 0.1% genistein). Upper panel illustrates a 1 h section of the 8 h EPG. Lower panels present the details of individual EPG waveforms corresponding with the display in the upper panel. ‘G’—stylets in the xylem (EPG waveform visualizes the active uptake of xylem sap); ‘C’—stylets in epidermis and mesophyll (EPG waveform visualizes the progressive stylet movements within the apoplast and occasional uptake of sap from cells adjacent to the stylet track represented as potential drops ‘pd’); ‘E1’—stylets in phloem (egestion of saliva into sieve elements); ‘E2’—stylets in phloem (passive ingestion of phloem sap from sieve elements).

Aphid activities on plants, genistein-treated and untreated, embraced two major phases: no-probing, when aphid stylets were outside of the plant tissues, and probing, when aphid mouthparts were actively progressing within the plant. Aphid probing was divided into the pathway, xylem, and phloem phases, which corresponded with the position of stylets in mesophyll, xylem vessels, and sieve elements in the phloem, respectively (Figure 2). The EPG-recorded aphid probing was evaluated in terms of general aspects that summarize various activities and trends in aphid behavior and in terms of the sequence of crucial events in individual phases of stylet penetration in plant tissues.

### 2.1. Impact of Genistein on Aphid Probing Behavior: General Aspects

**Figure 3:**
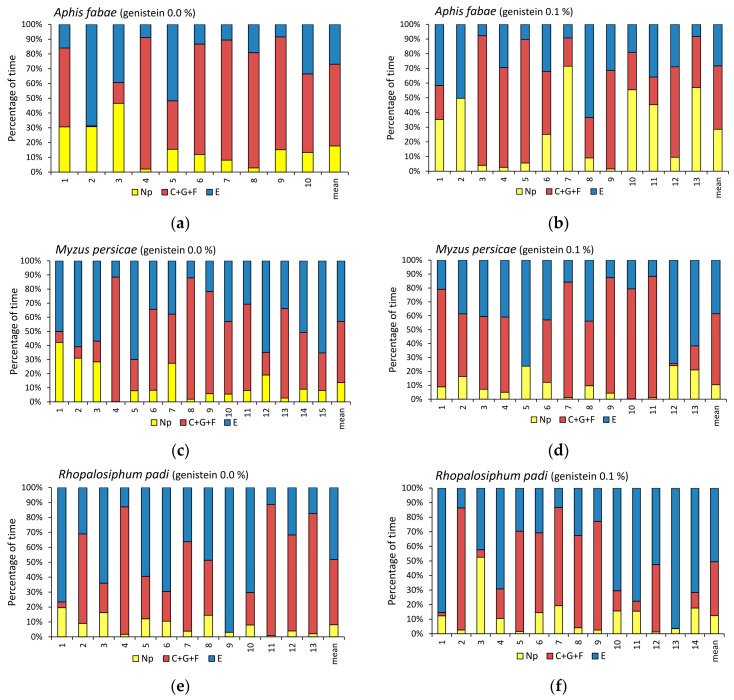
Individual variation in probing behavior of aphids on control untreated plants and plants treated transepidermally with 0.1% genistein: (**a**,**b**) *Aphis fabae* on *Vicia faba*; (**c**,**d**) *Myzus persicae* on *Brassica rapa* ssp. *pekinensis;* and (**e**,**f**) *Rhopalosiphum padi* on *Avena sativa*. Panels (**a**–**f**) represent the proportion of time (percentage of cumulative time for individual aphids and the mean of the group) devoted to Np—no probing, C + F + G—pathway + derailed stylet activities + xylem phase, and E—phloem phase E1 (salivation) + E2 (sap ingestion) activities recorded during the 8 h EPG experiments.

**Figure 4:**
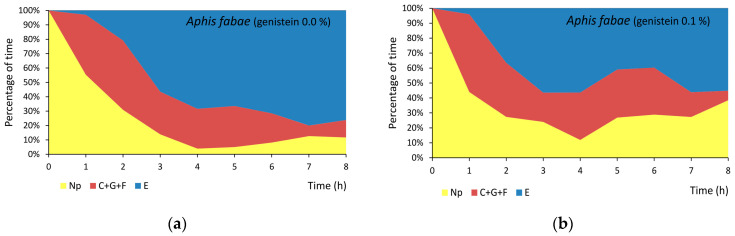

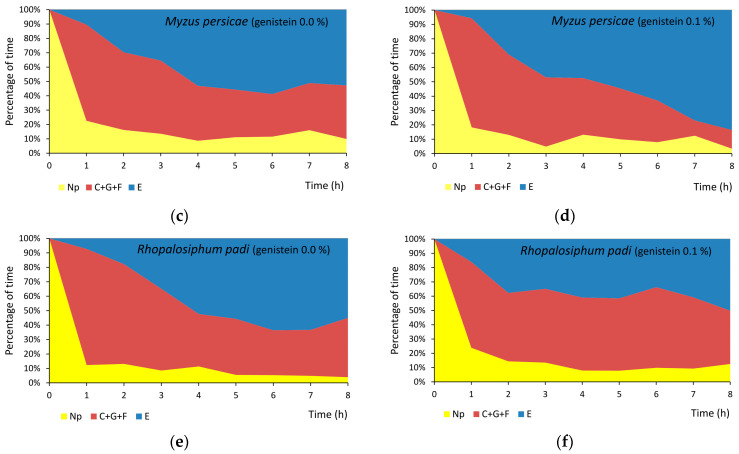
Sequential changes in EPG-recorded stylet penetration activities of aphids on control untreated plants and plants treated transepidermally with 0.1% genistein: (**a**,**b**) *Aphis fabae* on *Vicia faba*; (**c**,**d**) *Myzus persicae* on *Brassica rapa* ssp. *pekinensis*; and (**e**,**f**) *Rhopalosiphum padi* on *Avena sativa*. Panels (**a**–**f**) represent the proportion of time (average percentage of cumulative time for aphids in the group) devoted to Np—no probing, C + F + G—pathway + derailed stylet activities + xylem phase, and E—phloem phase E1 (salivation) + E2 (sap ingestion) activities during the consecutive hours of 8 h EPG recording.

Generally, in all studied aphid–plant treatment combinations, a noticeable individual variation in aphid behavior occurred, which resulted in the differences in the proportion of the total durations of three major behavioral phases: no-probing, probing in non-phloem tissues, and probing in the phloem (Figure 3a–f). However, the proportion, duration, and frequency of particular activities were specific for each aphid–genistein combination (Figure 4a–f, Table 1 and Table S1).

In *Aphis fabae*, the ratio of no-probing-to-probing activities was 1:4.6 and 1:2.5 on untreated and 0.1% genistein-treated plants, respectively. On genistein-treated plants, the maximum duration of a no-probing period was 2.3 times longer than on untreated plants (Table 1). The decline in probing activities in favor of no probing took place from the 4th hour until the end of the 8 h experiment (Figure 4a,b). The total and mean durations of pathway activities were similar in all aphids. The derailment of stylets during pathway (‘F’) occurred sporadically and only on plants treated with genistein. The xylem phase (‘G’) was rarely observed (Table 1 and Table S1). The total duration of the phloem phase (‘E1’ + ‘E2’) was slightly (1.3 times) shorter on genistein-treated plants than on untreated plants. In more detail, the values of parameters describing the salivation (‘E1’) segment of the phloem phase were similar in aphids on treated and untreated plants, but there was a trend towards the reduction in the duration of phloem sap ingestion (‘E2’) on genistein-treated plants. Although the number and mean duration of sap ingestion bouts were similar in all aphids, the duration of the first sap ingestion period was 1.5 times shorter, and the proportion of sustained sap ingestion periods to all probing activities was 7% lower on genistein-treated plants (Table 1 and Table S1).

In *Myzus persicae*, the ratio of no-probing-to-probing activities was 1:6.4 and 1:8.6 on untreated and 0.1% genistein-treated plants, respectively. On genistein-treated plants, the maximum duration of a no-probing period was 1.7 times shorter than on untreated plants (Table 1 and Table S1). The decline in no probing activities in favor of probing in the studied group of aphids took place from the 2nd hour onwards with a predominance of the phloem phase at the end of the 8 h experiment (Figure 4c,d). As a whole, however, the total and mean durations of pathway activities were similar in all aphids during the 8 h experiment. The derailment of stylets during pathway (‘F’) occurred sporadically on both the treated and untreated plants but significantly more frequently on untreated plants. The xylem phase (‘G’) was rarely observed (Table 1). The total duration of the phloem phase (‘E1’ + ‘E2’) was slightly (1.3 times) longer on genistein-treated plants than on untreated plants. The values of parameters describing the salivation (‘E1’) segment of the phloem phase were generally similar in aphids on treated and untreated plants, but the total duration of ‘E1’ followed by sustained ingestion (‘E2’ longer than 10 min) was significantly 2.1 times shorter in aphids on genistein-treated plants. The number and mean duration of sap ingestion bouts were similar in all aphids, but the duration of the first sap ingestion period was 1.2 times longer, and the proportion of sustained sap ingestion periods to all probing activities was 11% higher on genistein-treated plants (Table 1 and Table S1).

In *Rhopalosiphum padi*, the ratio of no-probing-to-probing activities was 1:11.3 and 1:7.1 on untreated and 0.1% genistein-treated plants, respectively. On genistein-treated plants, the maximum duration of a no-probing period was 1.8 times longer than on untreated plants (Table 1 and Table S1). The relatively low proportion of sap ingestion at approximately 30% of all activities occurred from the 2nd hour until the end of the 8 h experiment. At the same time, the proportion of no probing remained low. The main activity of aphids on genistein-treated plants was pathway, while on untreated plants, the main activity was phloem sap ingestion (Figure 4e,f). The total duration of the phloem phase (E) was similar in genistein-treated and untreated plants, but the contribution of salivation (‘E1’) was 9% higher in genistein-treated plants. The mean durations of sap ingestion (‘E2’) and sustained sap ingestion (‘E2’ > 10 min.) were 2.2 and 1.8 times shorter on genistein-treated plants than in untreated plants. The derailment of stylets during pathway (‘F’) and the xylem phase (‘G’) occurred relatively frequently, and the durations of these activities were similar on both the treated and untreated plants (Table 1 and Table S1).

### 2.2. Pre-Ingestive and Ingestive Phases of Aphid Probing: Sequence of Events

Sequential analysis of aphid probing presented in this study embraces various events within six subdivisions of the EPG recordings: the period before the first phloem phase (i.e., before the first contact with phloem sieve elements), the first phloem phase, the period before the first sap ingestion phase, the period before the first sustained sap ingestion phase (‘E2’ longer than 10 min), the period after the first phloem phase, and the period after the first sustained sap ingestion phase (Table 2 and Table S2).

Generally, statistical analysis did not show significant differences in the values of EPG parameters describing the behavior of aphids on untreated and genistein-treated plants. However, certain tendencies were noticeable.

**Figure 5:**
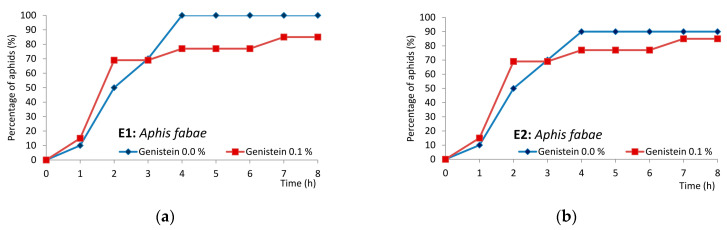

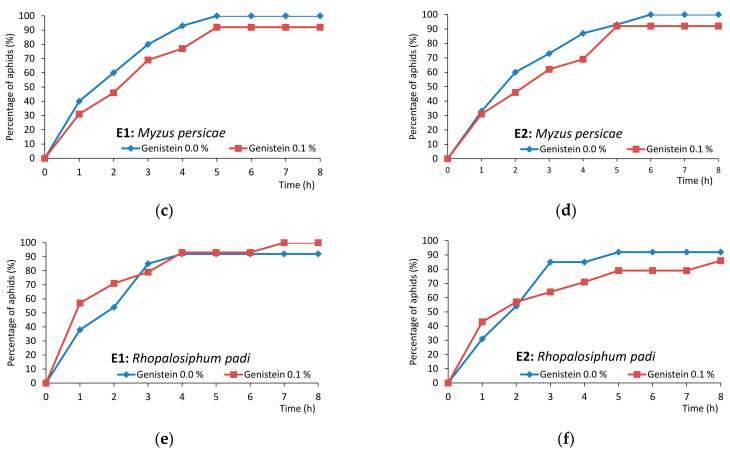
Cumulative percentage of aphids that attained phloem phase (=reached sieve elements) during the 8 h EPG monitoring on control untreated plants and plants treated transepidermally with 0.1% genistein: (**a**,**b**) *Aphis fabae* on *Vicia faba*; (**c**,**d**) *Myzus persicae* on *Brassica rapa* ssp. *pekinensis*; and (**e**,**f**) *Rhopalosiphum padi* on *Avena sativa*. E1—phloem phase salivation represents any contact with sieve elements; E2—phloem phase sap ingestion represents actual feeding, i.e., the uptake of phloem sap.

In *Aphis fabae*, the first probe was nearly 40 times shorter, the no-probing period before the first phloem phase was 1.3 times longer, and the first phloem phase was 1.6 times shorter on genistein-treated plants than on untreated plants (Table 2 and Table S2). All aphids of the studied population on untreated plants reached phloem vessels within the first four hours after access to plants, while on genistein-treated plants, 20% of aphids failed to locate sieve elements within the 8 h experiment (Figure 5a). On untreated plants, 90% of aphids commenced sap ingestion, while on genistein-treated plants—80% (Figure 5b).

In *Myzus persicae*, the first probe was nearly nine times shorter, the time from the first probe to the first phloem phase and first sustained ingestion phase was 1.4 and 1.3 times longer, and the time to reach the first phloem phase and first ingestion phase within the probe was 1.9 and 2.1 times longer on genistein-treated plants than on untreated plants. The number of new probes and short probes after the first phloem phase was 1.8 times lower on genistein-treated plants (Table 2 and Table S2). On untreated plants, 90% of aphids reached the phloem phase and started ingestion, while on genistein-treated plants—85%. The timing of the location of sieve elements and the commencement of ingestion was similar in aphids on untreated and genistein-treated plants (Figure 5c,d).

In *Rhopalosiphum padi*, the first probe was 1.8 times shorter, the time from the first probe to the first phloem phase was 1.5 times shorter, the no-probing period before the first phloem phase was 2.1 times longer, and the first phloem phase was 2.3 times shorter on genistein-treated plants than on untreated plants (Table 2 and Table S2). On untreated plants, 85% of aphids reached sieve elements and started ingestion within the first four hours after access to plants; finally, 92% of aphids reached ingestion within eight hours. On genistein-treated plants, 90% of aphids reached sieve elements, and 71% started ingestion within four hours from the beginning of the experiment. Finally, all aphids reached sieve elements, but 86% of them started ingestion (Figure 5e,f).

## 3. Discussion

The results of the present research indicate that transepidermal application of genistein to the host plants did not affect the probing behavior of the black bean aphid, the peach–potato aphid, and the bird cherry-oat aphid in spectacular ways. The poor response of aphids to genistein observed in the present study might have resulted from several reasons. First, the high degree of polyphagy of the aphid species studied might suggest that the sensitivity to genistein is inherently low in these aphids. Second, the method of compound application might have caused an uneven distribution of the chemical in the plant, and/or the compound might have been metabolized within the plant, thus becoming unavailable to aphid gustatory receptors. Finally, the high individual variability in behavior within the studied aphid populations might have affected the results of the statistical analysis.

As for the first issue specified earlier, all studied aphid species, the black bean aphid, the peach–potato aphid, and the bird cherry-oat aphid, belong to the group that under natural conditions of the cold temperate climate alternates between the so-called ‘winter’ hosts (=primary hosts) and ‘summer’ hosts (=secondary hosts), depending on the season. Primary hosts are plants, usually trees or shrubs, where these aphids reproduce bisexually and overwinter, while secondary hosts are these plants, usually herbaceous, on which aphids reproduce parthenogenetically during the vegetative—‘summer’—season [28,29]. The ranges of the summer hosts of the studied species are very broad and include crop plants of worldwide importance. The black bean aphid overwinters mainly on spindle tree *Euonymus europaeus* L. (Celastraceae), but in the spring it colonizes a wide range of hosts, including young growth of some trees and many crops, chiefly of the Fabaceae family [29]. The peach–potato aphid overwinters mainly on peach *Prunus persica* (L.) Batsch (Rosaceae), but the range of its secondary hosts is extremely broad, including plants of more than 40 botanical families [29]. The primary host of the bird cherry-oat aphid is mainly bird cherry, *Prunus padus* L. (Rosaceae), in Europe and common choke cherry, *Prunus virginiana* L. (Rosaceae), in North America, but during summer, it attacks all the major cereals and pasture grasses [28,29].

Genistein is considered one of the most common natural flavonoids in the plant kingdom. Although it is sourced mainly from soybean, it is widely distributed in other leguminous plants such as alfalfa *Medicago sativa* L. and clover *Trifolium* spp. L. [42,43]. Various amounts of genistein have been reported also in plants other than Fabaceae, e.g., cranberry *Vaccinium oxycoccos* L. (Ericaceae), asparagus *Asparagus officinalis* L. (Asparagaceae), raspberry *Rubus idaeus* L. (Rosaceae), spinach *Spinacia oleracea* L. (Amaranthaceae), lettuce *Lactuca sativa* L. (Asteraceae), rice *Oryza sativa* L. (Poaceae), cucumber *Cucumis sativus* L. (Cucurbitaceae), date *Phoenix dactylifera* L. (Araceae), and many others [42,43,44,45,46]. The ubiquity of genistein in such a variety of plant species suggests that polyphagous herbivores such as the studied aphids may come into contact with this flavonoid while exploring the plants and feeding. Hence, it is likely that the three aphid species studied have evolved a tolerance to genistein in their diet under natural conditions. We found similar weak effects of quercetin and rutin on aphid behavior: while quercetin was either inactive behaviorally or weakly stimulatory for probing activities of *A. pisum*, *M. persicae*, and *R. padi* on their respective host plants, rutin evoked significant negative responses in *A. pisum* and *M. persicae*, but not in *R. padi* [38]. Apigenin, daidzein, and kaempferol caused a decrease in the duration of plant sap ingestion by *A. pisum*, but genistein did not affect the feeding activity of this aphid [27]. The background of relative tolerance of aphids to various flavonoids may be related to the omnipresence of flavonoids in plants, which is a consequence of the crucial roles these compounds play in plant metabolism and environmental interactions [47,48].

Another issue is the method we used to assess the impact of genistein on aphid behavior. There are three basic ways to evaluate the effect of a chemical on aphid probing behavior: (i) application in a complete artificial diet, a gel, or a sucrose diet; (ii) application in the feeding medium for direct uptake via the plant vascular system; and (iii) transepidermal application to plants by dipping or spraying. The first method allows the verification of the compound’s activity [25]. The second and the third methods allow the verification of both the activity and the compound transport within the plant. Using these methods in combination with electropenetrography, it is also possible to evaluate the compound’s activity in particular plant tissues and, in fact, assess the fate of the compound within the plant [37,41,49,50,51]. Aphid behavioral responses to allelochemicals in individual plant tissues make them ‘living sensors’ of the presence and activity of the studied compounds [37,50]. As the transepidermal treatment relates also to the practical aspects of potential application to aerial parts of the plants under field or greenhouse conditions, we apply this approach in our studies on allelochemical–aphid relationships [26,27,38].

In the present study, we determined that exogenous application of 0.1% genistein to plants did not affect the behavior of aphids prior to the insertion of stylets in any of the studied species on their respective host plants: all aphids initiated probing. Subsequently, during the period before the first phloem phase, when the effect of the sieve element sap on aphid behavior could be excluded, aphids withdrew their stylets and initiated new probes with similar frequency on both untreated and genistein-treated plants. The time to reach sieve elements within a successful probe was similar in aphids on treated and untreated plants in each aphid species combination. The assessment of plant chemistry in the course of probing through plant non-vascular tissues is the crucial phase in the host plant selection process by aphids [30,51]. In view of the present results, genistein is not an inhibitor of aphid probing activities in non-phloem tissues. During the phloem phase, which follows the non-phloem phase in the sequence of probing activities, aphids ingest the sap for nutritive purposes and concurrently secrete saliva into the sieve elements to suppress plant defense responses [30]. Hence, the duration of sap ingestion is a demonstration of the amount of the consumed food and plant suitability, while the duration of salivation reflects the potency of plant defenses [30,52,53,54]. In the present study, the contribution of sap ingestion to overall probing was substantial in all aphids studied: it ranged from 43% in *R. padi* on genistein-treated oats to 68% in *A. fabae* on untreated beans. In *A. fabae* and *R. padi*, a slight reduction in sap ingestion occurred on genistein-treated plants. In contrast, in *M. persicae*, an increase in sap ingestion occurred on genistein-treated cabbage. These tendencies might indicate a potential inhibitory activity of genistein in *A. fabae* and *R. padi* and a potential stimulatory activity of genistein in *M. persicae* as far as phloem sap consumption is concerned. The contribution of salivation to the phloem phase ranged from 1.8% in *A. fabae* on genistein-treated plants to 23% in *R. padi* on genistein-treated plants. A very high percentage of phloem salivation was observed in aphids confronted with various deterrent chemicals and non-host plants [48,55,56,57]. It is likely then that genistein has a deleterious effect on *R. padi*. This deduction is supported also by the overall analysis of aphid behavior: the ratios of probing to no-probing activities during the ‘pre-phloem’ period were in favor of probing on untreated plants in *A. fabae* and *R. padi* and on treated plants in *M. persicae*, and the proportion of time devoted to sap consumption decreased in *A. fabae* and *R. padi* and increased in *M. persicae* in the course of the 8 h of monitoring.

Finally, the lack of statistically significant differences between the values of parameters describing aphid behavior on untreated and genistein-treated plants might have been due to the high variability. It is generally accepted that variability can reduce the statistical power of a test [58,59,60,61]. In our experiments, high variability within aphid populations occurred irrespective of the treatment. Such a phenomenon is typical for studies on aphid probing behavior. Nevertheless, it depends solely on the potency of the compound whether the statistical analysis reveals alterations in aphid behavior on the treated plants. Using a similar method of compound application, we confirmed this statement in our previous studies [49,56,62,63]. Therefore, we are of the opinion that the treatment of plants with genistein at 0.1% concentration does not effectively alter the probing behavior of the polyphagous aphids studied, despite the reported trend of reducing sap ingestion in *A. fabae* and *R. padi* and stimulating ingestion in *M. persicae* on treated plants. The function of genistein as a generalist herbivore deterrent has also been ruled out in the study involving the gypsy moth, the polyphagous tree folivore [17]. Although it is possible that the use of higher concentrations of genistein generates stronger behavioral effects in generalist aphids, as in the case of the oligophagous *A. pisum* [25], it seems unpractical from the potential field application perspective.

## 4. Materials and Methods

### 4.1. Cultures of Plants and Aphids

Laboratory cultures of *Aphis fabae*, *Myzus persicae*, and *Rhopalosiphum padi* were kept on *Vicia faba* L. cv. Windsor Biały, *Brassica rapa* ssp. *pekinensis* (Lour.) Hanelt cv. Hilton, and *Avena sativa* L. cv. Komfort, respectively, in the laboratory of the Department of Botany and Ecology, University of Zielona Góra, Poland. Both aphids and plants were kept under the same laboratory conditions, i.e., at 20 °C, 65% r.h., and L16:8D photoperiod. Four- to seven-day-old apterous aphid females and three-week-old plants with 4–5 fully developed leaves were used for the experiments. Plants used for experiments were the same plant species and cultivars that were used for the rearing of aphids.

### 4.2. Application of Genistein

Genistein was purchased from Sigma-Aldrich (Poznań, Poland). The flavonoid was dissolved in 70% ethanol to obtain a 0.1% solution. To simulate the natural environment under laboratory conditions, genistein was presented to aphids by application through their host plants. For our study, genistein was applied on the adaxial and abaxial leaf surfaces by immersing one leaf of an intact plant in the ethanolic solution of 0.1% concentration for 30 s. Preparation and application of the compounds followed the procedure described by Polonsky et al. [64], later modified by Gabryś et al. [65]. Control leaves were immersed in 70% ethanol that was used as a solvent for the studied compound. There is no effect of ethanol application on aphid probing behavior and plant condition [66]. All experiments were performed 1 h after the compound application to allow for the evaporation of the solvent.

### 4.3. Aphid Probing Behavior (No-Choice Experiment)

Aphid probing behavior was studied using the electrical penetration graph (=electropenetrography, EPG) technique. This experimental system allows monitoring of the feeding behavior of piercing–sucking insects, such as aphids, during their stylet penetration in plant tissues in real-time [30,67,68]. Using the EPG technique, it is possible to reveal the effect of exogenously applied compounds that may influence plant–aphid interactions [27,38,40,41]. The EPG is based on the incorporation of the golden-wire-tethered insects by means of conductive water-based silver paint into the electrical circuit that also includes plants that are their food source. The electrical circuit is completed when the aphid inserts its stylets into the plant [67,68,69] (Figure S1). Weak voltage is supplied in the circuit, and all changing electric properties are recorded as EPG waveforms that can be correlated with aphid activities and stylet position in plant tissues [30,36]. Before starting the experiment, the aphids were starved for 1 h. The probing behavior of apterous females was monitored for 8 h continuously with the eight-channel DC EPG recording equipment (EPG-Systems, Dillenburg 126,703 CJ Wageningen, The Netherlands). Recordings that terminated due to aphids falling from the plant or where the EPG signal was unclear were discarded from the analysis. All experiments were carried out under the same conditions of temperature, relative humidity (r.h.), and photoperiod as those used for the rearing of plants and aphids. All bioassays started at 10:00–11:00 h MEST (Middle European Summer Time). Joschinski et al. [70] found that aphids have diurnal rhythms even on constant food sources and are more active during the day than during the night.

Electrical penetration graphs were acquired and analyzed using the PROBE 3.1 software provided by Dr. W.F. Tjallingii (https://www.epgsystems.eu; Wageningen 6703 CJ, The Netherlands). The following waveform patterns were identified: no penetration (waveform ‘np’—aphid stylets outside the plant) and pathway phase—penetration of non-phloem tissues (waveforms ‘ABC’), derailed stylet movements (waveform ‘F’), salivation into sieve elements (waveform ‘E1’), ingestion of phloem sap (waveform ‘E2’), and ingestion of xylem sap (waveform ‘G’). The waveform patterns that were not terminated before the end of the experimental period (8 h) were included in the calculations. Only the replications that included complete 8 h recordings were kept for analysis. The final number of the analyzed EPG recordings (=the number of replications) were *Aphis fabae*—10 (genistein 0.0%) and 12 (genistein 0.1%), *Myzus persicae*—15 (genistein 0.0%) and 13 (genistein 0.1%), and *Rhopalosiphum padi*—14 (genistein 0.0%) and 13 (genistein 0.1%). The parameters derived from EPGs were analyzed according to their frequency and duration in a configuration related to activities in peripheral and vascular tissues. All EPG parameters describing aphid probing behavior were calculated using the Novel Program for Automatic Calculation of EPG Variables v1.0 (2024) [71]. Subsequently, the means and standard deviations were determined. Aphid behavior on leaves treated with genistein was compared to aphid behavior on control plants. A two-sample Student’s *t*-test was used for these comparisons. All analyses were performed using Genstat v. 23.1 statistical software [72].

## Figures and Tables

**Table 1:** Probing behavior of *Aphis fabae*, *Myzus persicae*, and *Rhopalosiphum padi* recorded in the EPG no-choice test on host plants treated transepidermally with 0.1% ethanolic solutions of genistein: non-sequential EPG parameters.

EPG Variable	*Aphis fabae*		*Myzus persicae*		*Rhopalosiphum padi*	
	Genistein		Genistein		Genistein	
	0.0%	0.1%	0.0%	0.1%	0.0%	0.1%
No probing (Np)						
Total duration of Np	84.7 ± 67.8	137.2 ± 119.5	65.6 ± 61.0	50.0 ± 41.0	38.9 ± 29.3	59.4 ± 63.8
Number of Np	16.9 ± 16.4	13.7 ± 7.9	35.7 ± 25.6	28.0 ± 19.1	11.8 ± 8.1	13.0 ± 7.4
Mean duration of Np	7.7 ± 7.3	11.9 ± 14.8	1.8 ± 1.1	1.6 ± 0.8	3.4 ± 1.5	4.3 ± 3.5
Maximum Np	39.8 ± 45.0	90.6 ± 105.6	15.0 ± 31.0	8.7 ± 9.7	11.0 ± 7.6	20.3 ± 27.9
Probing (C + F + G + E)						
Total probing time	395.3 ± 67.8	342.8 ± 119.5	414.4 ± 61.0	430.0 ± 41.0	441.1 ± 29.3	420.6 ± 63.4
Mean duration of probes	83.3 ± 141.4	46.2 ± 60.1	49.0 ± 120.1	37.6 ± 45.5	63.4 ± 45.6	49.8 ± 37.9
Number of probes	16.8 ± 16.5	13.3 ± 7.8	35.7 ± 25.5	28.0 ± 19.1	11.7 ± 8.1	12.9 ± 7.5
Number of short probes (<3 min)	10.8 ± 9.6	7.9 ± 5.6	23.5 ± 19.6	17.0 ± 13.5	5.1 ± 4.7	5.5 ± 4.2
Pathway phase (C)						
Total duration of C	124.6 ± 95.2	130.8 ± 75.9	173.5 ± 86.7	145.0 ± 83.7	108.7 ± 62.9	128.9 ± 64.5
Number of C	18.4 ± 16.4	15.1 ± 8.7	39.5 ± 26.5	31 ± 19.6	21.0 ± 11.5	23.0 ± 11.4
Mean duration of C	8.4 ± 4.3	10.5 ± 8.9	8.5 ± 13.2	5.3 ± 1.9	5.2 ± 2.5	5.8 ± 1.6
Proportion of probing spent in C (%)	34.9 ± 30.9	40.5 ± 23.6	44.9 ± 26.7	36.0 ± 23.3	25.6 ± 16.0	31.3 ± 15.7
Derailed stylet activities (F)						
Total duration of F	0 ± 0	1.7 ± 6.1	21.1 ± 30.2	8.8 ± 25.1	68.4 ± 57.6	55.2 ± 68.6
Number of F	0 ± 0	0.1 ± 0.2	**0.8 ± 0.4**	**0.2 ± 0.4**	3.7 ± 2.4	2.8 ± 4.0
Mean duration of F	0 ± 0	0 ± 0	0 ± 0	0 ± 0	21.3 ± 12.6	23.2 ± 12.6
Proportion of probing spent in F (%)	0 ± 0	0 ± 0	0 ± 0	0 ± 0	17.5 ± 14.2	22.1 ± 14.8
Xylem phase (G)						
Total duration of G	5.0 ± 12.6	3.2 ± 8.7	11.4 ± 21.7	31.0 ± 37.5	54.0 ± 117.0	58.9 ± 65.4
Number of G	0.2 ±0.4	0.2 ± 0.6	0.9 ± 1.9	0.8 ± 0.6	1.0 ± 2.2	1.9 ± 2.4
Mean duration of G	0 ± 0	0 ± 0	14.9 ± 9.5	41.0 ± 38.0	101.1 ± 104.0	44.8 ± 31.5
Proportion of spent in G (%)	0 ± 0	0 ± 0	11.0 ± 10.1	8.3 ± 5.5	0 ± 0	0 ± 0
Phloem phase: general (E = E1 + E2)						
Total duration of phloem phase E (E1 + E2)	294.0 ± 120.4	224.0 ± 123.2	208.0 ± 132.3	265.0 ± 130.8	219.0 ± 145.0	197.0 ± 151.0
Total duration of E1	9.4 ± 12.1	3.3 ± 2.6	2.4 ± 1.8	3.7 ± 2.1	16.6 ± 22.0	12.7 ± 12.4
Total duration of E2	284.8 ± 121.0	221 ±123.2	206.6 ± 133.4	262.0 ± 131.0	211.0 ± 148.0	192.4 ± 150.7
Phloem phase: salivation (E1)						
Number of E1	2.9 ± 2.0	2.3 ± 2.1	3.0 ± 2.1	2.8 ± 2.0	6.6 ± 4.6	7.4 ± 6.0
Mean duration of E1	2.6 ± 2.0	1.4 ± 0,9	0.8 ± 0.2	1.5 ± 1.2	2.0 ± 2.2	2.0 ± 2.0
Number of single E1	0.5 ± 0.8	0.3 ± 0.6	0.3 ± 0.5	0.5 ± 0.9	2.6 ± 2.4	2.6 ± 2.6
Total duration of E1 followed by E2	5.2 ± 5.4	2.3 ± 1.4	1.9 ± 1.2	2.5 ± 1.2	4.7 ± 4.3	3.6 ± 1.7
Total duration of E1 followed by E2 > 10 min	4.0 ± 4.8	2.0 ± 1.3	**2.1 ± 1.1**	**1.0 ± 0.5**	5.3 ± 5.7	2.0 ± 1.0
Contribution of E1 to phloem phase (%)	4.7 ± 5.5	1.8 ± 1.5	3.0 ± 3.8	8.0 ± 21.6	14.3 ± 17.3	23.1 ± 30.9
Proportion of probing spent in E1 (%)	2.3 ± 2.7	1.0 ± 0.8	0.6 ± 0.5	0.9 ± 0.6	3.9 ± 5.3	3.2 ± 3.1
Phloem phase: sap ingestion (E2)						
Number of E2	2.2 ± 1.7	1.8 ± 1.4	2.5 ± 1.6	2.2 ± 1.6	2.8 ± 1.6	3.6 ± 3.0
Number of E2 > 10 min	1.3 ± 0.7	1.4 ± 0.8	1.5 ± 0.7	1.6 ± 1.1	1.2 ± 0.7	1.5 ± 1.2
Mean duration of E2	184.5 ± 150.0	152.0 ± 113.2	147.3 ± 142.9	150.0 ± 126.4	126.0 ± 140.0	58.6 ± 51.2
Duration of the longest E2	266.4 ± 133.5	194.0 ± 116.3	183.4 ± 136.0	216.0 ± 124.0	187.4 ± 151.0	146.5 ± 117.0
Duration of the 1st E2	184.0 ± 183.0	119.2 ± 125.0	136.8 ± 145.5	117.0 ± 142.5	129.8 ± 175.0	46.5 ± 84.3
Total duration of E2 > 10 min	254.4 ± 146.0	202.0 ± 134.0	202.1 ± 137.4	239.5 ± 144.5	192.1 ± 154.8	160.2 ± 155.8
Mean duration of E2 > 10 min	227.0 ± 142.0	161.4 ± 105.5	171.4 ± 137.5	187.0 ± 114.2	195.3 ± 150.5	108.4 ± 86.9
Proportion of E2 > 10 min	95.2 ± 46.1	86.9 ± 19.7	71.8 ± 32.5	78.9 ± 31.9	52.2 ± 34.3	58.2 ± 34.7
Proportion of probing spent in E2 (%)	68.4 ± 24.7	61.5 ± 20.6	46.9 ± 27.1	58.0 ± 27.0	46.3 ± 31.0	43.4 ± 33.3

Durations of various stylet activities are given in minutes (mean ± SD). **Bold** indicates values that are significantly different between treatments at *p* < 0.05 (ANOVA; details in Table S1).

**Table 2:** Probing behavior of *Aphis fabae*, *Myzus persicae*, and *Rhopalosiphum padi* recorded in the EPG no-choice test on host plants treated transepidermally with 0.1% ethanolic solutions of genistein: sequential EPG parameters.

EPG Variable ^1^	*Aphis fabae*		*Myzus persicae*		*Rhopalosiphum padi*	
	Genistein		Genistein		Genistein	
	0.0%	0.1%	0.0%	0.1%	0.0%	0.1%
Before 1st phloem phase						
Duration of 1st probe	47.4 ± 148.6	1.2 ± 1.4	32.9 ± 123.5	3.6 ± 7.4	62.2 ± 119.1	34.9 ± 77.3
Time from 1st probe to 1st E	126.9 ± 58.5	151.0 ± 137.3	107.9 ± 74.5	155.4 ± 133.3	122.6 ± 123.8	82.0 ± 102.3
Time from the beginning of that probe to 1st E	34.9 ± 23.6	36.0 ± 24.8	22.3 ± 13.8	43.3 ± 38.2	36.0 ± 51.0	27.4 ± 29.4
Number of probes to the 1st E1	13.0 ± 10.1	10.1 ± 5.2	16.8 ± 13.2	18.0 ± 17.1	4.2 ± 2.2	4.1 ± 3.4
Number of short probes before 1st E	9.4 ± 7.5	6.3 ± 4.4	11.7 ± 10.3	11.2 ± 12.5	1.8 ± 1.6	1.9 ± 2.4
Duration of nonprobe period before the 1st E	60.0 ± 38.3	78.0 ± 105.2	24.1 ± 26.4	29.7 ± 33.4	12.0 ± 6.9	25.0 ± 47.1
1st phloem phase						
Duration of 1st phloem phase E	184.8 ± 181.5	112.4 ± 129.0	133.4 ± 148.6	116.6 ± 144.2	86.6 ± 162.8	38.2 ± 80.1
Before 1st sap ingestion phase E2						
Time from 1st probe to 1st E2	163.4 ± 121.7	162.8 ± 149.0	123.9 ± 99.1	162.7 ± 135.8	142.0 ± 129.1	157.5 ± 175.5
Time from the beginning of that probe to 1st E2	44.9 ± 27.7	38.2 ±25.8	21.9 ± 13.9	45.6 ± 37.9	24.2 ± 13.7	25.6 ± 20.4
Number of probes before 1st E2	15.3 ± 16.6	10.1 ± 5.3	19.7 ± 19.1	18.5 ± 17.2	4.8 ± 2.8	6.1 ± 5.2
Before 1st sap ingestion phase E2 > 10 min						
Time from 1st probe to 1st E2 > 10 min	186.6 ± 131.2	179.0 ± 149.0	157.9 ± 117.3	185.4 ± 150.8	247.1 ± 151.0	207.7 ± 181.7
Time from the beginning of that probe to 1st E2 > 10 min	42.8 ± 29.2	40.1 ± 26.6	28.0 ± 14.0	38.2 ± 23.1	27.2 ± 12.2	32.0 ± 22.4
Number of probes before 1st sustained E2	15.7 ± 16.5	11.4 ± 6.7	22.8 ± 21.8	20.6 ± 17.7	8.6 ± 7.5	9.4 ± 7.6
After 1st phloem phase						
Number of probes after 1st E	3.9 ± 7.5	3.5 ± 6.6	18.9 ± 19.2	10.6 ± 12.1	8.2 ± 7.0	8.7 ± 6.7
Number of probes shorter than 3 min after 1st E	1.4 ± 2.5	1.7 ± 4.2	11.9 ± 14.1	6.6 ± 8.1	3.5 ± 3.5	3.3 ± 2.7
Potential E2 index	80.4 ± 30.5	70.0 ± 31.4	45.0 ± 26.7	35.7 ± 23.3	56.1 ± 33.3	56.1 ± 36.5
After 1st sap ingestion phase E2 > 10 min						
Number of probes after 1st sustained E2	1.2 ± 2.4	2.1 ± 3.8	12.9 ± 17.5	8.5 ± 12.0	2.7 ± 3.5	5.5 ± 5.8

^1^ Durations of various stylet activities are given in minutes (mean ± SD).

## Data Availability

All original data are available from authors upon request.

## Supplementary Materials

The following supporting information can be downloaded at: https://www.mdpi.com/article/10.3390/molecules29235715/s1, Table S1: Statistical analysis of the probing behavior of *Aphis fabae*, *Myzus persicae*, and *Rhopalosiphum padi* recorded in the EPG no-choice test on host plants treated transepidermally with 0.1% ethanolic solutions of genistein: non-sequential EPG parameters; Table S2: Statistical analysis of the robing behavior of *Aphis fabae*, *Myzus persicae*, and *Rhopalosiphum padi* recorded in the EPG no-choice test on host plants treated transepidermally with 0.1% ethanolic solutions of genistein: sequential EPG parameters; Figure S1: Experimental EPG system for recording of aphid probing behavior. A. Inside the Faraday cage: plants connected to plant electrodes and EPG probes connected to aphids. Outside the Faraday cage: Giga-8 DC EPG amplifier and voltage source. B. EPG probe connected to the aphid. C. Aphid with the attached golden wire electrode.
