# Supplementary material for: Genistein and Aphid Probing Behavior: Case Studies on Polyphagous Aphid Species
Source: Molecules. 2024 Dec 3;29(23):5715. doi: 10.3390/molecules29235715 (PMC11643683; doi:10.3390/molecules29235715)
Supplement: Supplementary file 1 [file molecules-29-05715-s001.zip › molecules-3332452-supplementary.pdf]

Table S1. Statistical analysis of the probing behavior of *Aphis fabae, Myzus persicae* and *Rhopalosiphum padi* recorded in the EPG no-choice test on host plants treated transepidermally with 0.1% ethanolic solutions of genistein: non-sequential EPG parameters.

**EPG variable**	***Aphis fabae***		***Myzus persicae***		***Rhopalosiphum padi***	
	***t*-Statistic**	***p*-value**	***t*-Statistic**	***p*-value**	***t*-Statistic**	***p*-value**
No probing						
Total duration of Np	1.24	0.229	-0.79	0.435	1.09	0.291
Number of Np	-0.57	0.58	-0.92	0.365	0.41	0.685
Mean duration of Np	0.89	0.384	-0.35	0.726	0.91	0.376
Maximum Np	1.56	0.137	-0.75	0.465	1.21	0.246
Probing						
Total probing time	-1,24	0,229	0.79	0.435	-1.09	0.291
Mean duration of probes	-0,77	0,458	-0.34	0.737	0.82	0.419
Number of probes	-0,62	0,548	-0.92	0.365	0.45	0.654
Number of short probes (< 3 min)	-0,9	0,378	-0.95	0.349	0.25	0.807
Pathway phase						
Total duration of C	0,17	0,865	-0.87	0.391	0.82	0.419
Number of C	-0,58	0,571	-1	0.326	0.45	0.654
Mean duration of C	0,74	0,472	-0.93	0.37	0.75	0.46
Proportion of probing spent in C (%)	0,49	0,63	-0.97	0.339	0.94	0.355
Derailed stylet activities						
Total duration of F	0,87	0,393	-1.16	0.256	-0.54	0.594
Number of F	0,87	0,393	-2.17	0.044	-0.7	0.489
Mean duration of F	-	-	-	-	0.27	0.787
Proportion of probing spent in F (%)	-	-	-	-	0.7	0.492
Xylem phase						
Total duration of G	-0,39	0,702	1.69	0.103	0.13	0.896
Number of G	0,14	0,892	-0.32	0.756	0.96	0.345
Mean duration of G	-	-	-	-	-1.06	0.36
Proportion of spent in G (%)	-	-	-	-	-	-
Phloem phase: general						
Total duration of phloem phase E (E1+E2)	-1,3	0,208	1.12	0.274	-0.37	0.716
Total duration of E1	-1,57	0,149	1.65	0.112	-0.57	0.576
Total duration of E2	-1,18	0,252	1.1	0.282	-0.3	0.765
Phloem phase: salivation (E1)						
Number of E1	-0,61	0,551	-0.2	0.845	0.36	0.724
Mean duration of E1	-1,66	0,122	1.87	0.086	0.05	0.962
Number of single E1	-0,62	0,539	0.72	0.481	-0.05	0.964
Total duration of E1 followed by E2	-1,55	0,156	1.18	0.251	-0.81	0.431
Total duration of E1 followed by E2 >10 min	-1,21	0,256	2.86	0.013	-1.92	0.082
Contribution of E1 to phloem phase (%)	-1,52	0,164	-1,52	0,164	-1,52	0,164
Proportion of probing spent in E1 (%)	-1,47	0,171	1.15	0.259	-0.43	0.674
Phloem phase: sap ingestion (E2)						
Number of E2	-0,55	0,589	-0,5	0,621	0,95	0,352
Number of E2 > 10 min	0,28	0,785	0,42	0,679	0,9	0,379
Mean duration of E2	-0,57	0,576	0,06	0,956	-1,56	0,141
Duration of the longest E2	-1,33	0,2	0,64	0,527	-0,74	0,465
Duration of the 1st E2	-0,96	0,347	-0,36	0,725	-1,49	0,157
Total duration of E2 > 10 min	-0,9	0,38	0,7	0,489	-0,53	0,599
Mean duration of E2 > 10 min	-1,21	0,24	0,31	0,762	-1,66	0,113
Proportion of E2 > 10 min	-0,5	0,626	1,21	0,238	0	0,999
Proportion of probing spent in E2 (%)	-0,7	0,495	1,08	0,289	-0,22	0,829

Table S2. Statistical analysis of the robing behavior of *Aphis fabae, Myzus persicae* and *Rhopalosiphum padi* recorded in the EPG no-choice test on host plants treated transepidermally with 0.1% ethanolic solutions of genistein: sequential EPG parameters.

**EPG variable**	***Aphis fabae***		***Myzus persicae***		***Rhopalosiphum padi***	
	***t*-Statistic**	***p*-value**	***t*-Statistic**	***p*-value**	***t*-Statistic**	***p*-value**
Before 1st phloem phase						
Duration of 1st probe	-0,98	0,351	-0,92	0,375	-0,71	0,483
Time from 1st probe to 1st E	0,56	0,581	1,14	0,27	-0,93	0,361
Time from the beginning of that probe to 1st E	0,1	0,918	1,81	0,093	-0,53	0,598
Number of probes to the 1st E1	-0,81	0,435	0,21	0,836	-0,01	0,992
Number of short probes before 1st E	-1,24	0,23	-0,1	0,92	0,2	0,843
Duration of nonprobe period before the 1st E	0,56	0,586	0,5	0,624	1,03	0,323
1st phloem phase						
Duration of 1st phloem phase E	-1,09	0,288	-0,3	0,77	-0,94	0,364
Before 1st sap ingestion phase E2						
Time from 1st probe to 1st E2	-0,01	0,992	0,87	0,391	0,26	0,797
Time from the beginning of that probe to 1st E2	-0,58	0,57	2,06	0,059	0,2	0,843
Number of probes before 1st E2	-0,94	0,368	-0,17	0,863	0,82	0,423
Before 1st sap ingestion phase E2 > 10 min						
Time from 1st probe to 1st E2 > 10 min	-0,13	0,9	0,54	0,591	-0,61	0,547
Time from the beginning of that probe to 1st E2 >10 min	-0,2	0,842	1,41	0,172	0,62	0,544
Number of probes before 1st sustained E2	-0,78	0,452	-0,29	0,775	-0,28	0,782
After 1st phloem phase						
Number of probes after 1st E	-0,13	0,895	-1,31	0,202	0,2	0,84
Number of probes shorter than 3 min after 1st E	0,23	0,821	-1,15	0,26	-0,18	0,861
Potential E2 index	-0,78	0,442	1,21	0,238	0	0,999
After 1st sap ingestion phase E2 > 10 min						
Number of probes after 1st sustained E2	0,6	0,559	-0,73	0,471	1,34	0,197

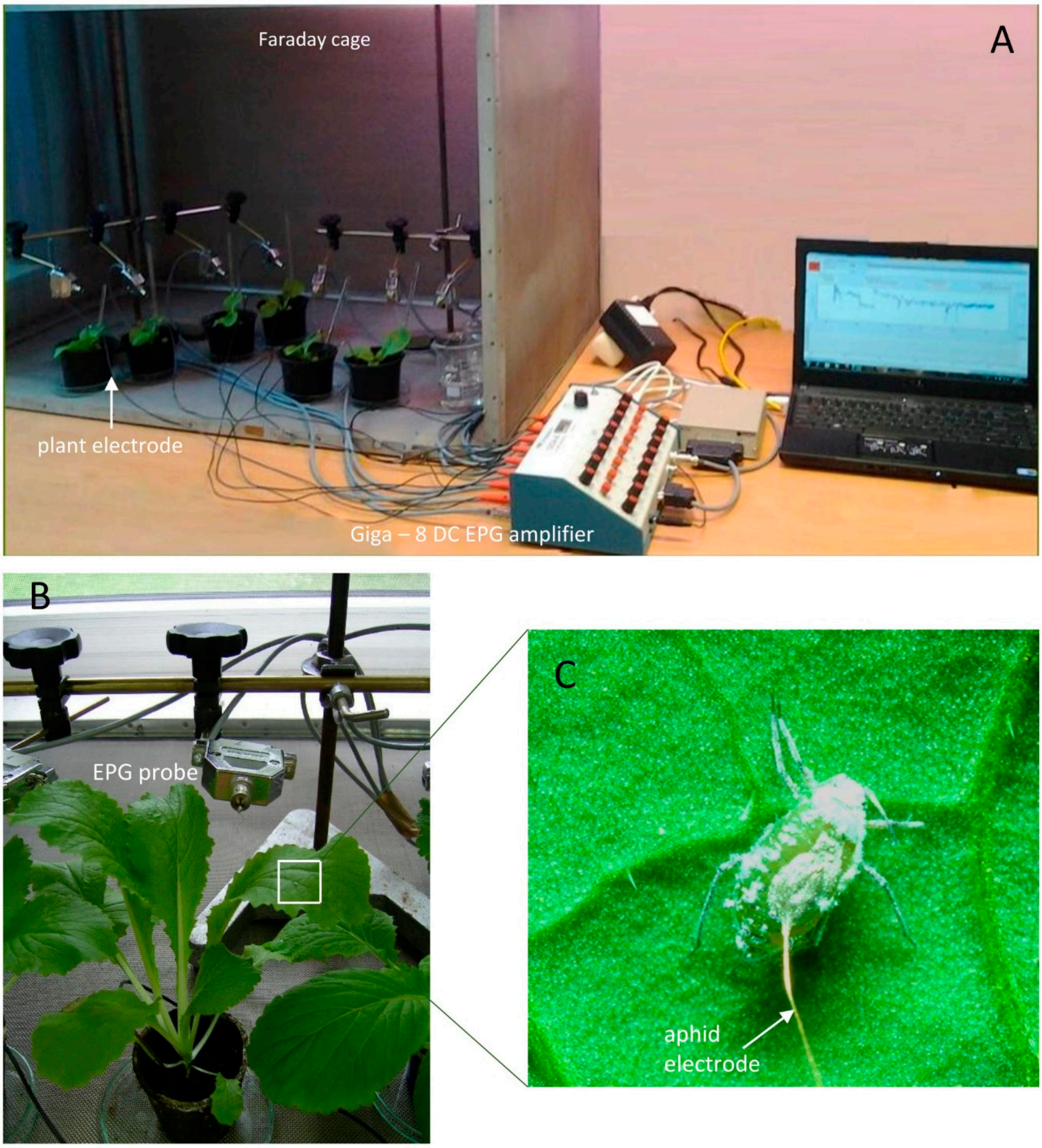

Figure S1: Experimental EPG system for recording of aphid probing behavior. A. Inside the Faraday cage: plants connected to plant electrodes and EPG probes connected to aphids. Outside the Faraday cage: Giga-8 DC EPG amplifier and voltage source. B. EPG probe connected to the aphid. C. Aphid with the attached golden wire electrode.
